# Three chromosomal rearrangements promote genomic divergence between migratory and stationary ecotypes of Atlantic cod

**DOI:** 10.1038/srep23246

**Published:** 2016-03-17

**Authors:** Paul R. Berg, Bastiaan Star, Christophe Pampoulie, Marte Sodeland, Julia M. I. Barth, Halvor Knutsen, Kjetill S. Jakobsen, Sissel Jentoft

**Affiliations:** 1 Centre for Ecological and Evolutionary Synthesis, Department of Biosciences, University of Oslo, N-0316 Oslo, Norway; 2 Marine Research Institute, Skúlagata 4, 101 Reykjavik, Iceland; 3 Institute of Marine Research, Flødevigen, N-4817 His, Norway; 4 Department of Natural Sciences, University of Agder, N-4604 Kristiansand, Norway

## Abstract

Identification of genome-wide patterns of divergence provides insight on how genomes are influenced by selection and can reveal the potential for local adaptation in spatially structured populations. In Atlantic cod – historically a major marine resource – Northeast-Arctic- and Norwegian coastal cod are recognized by fundamental differences in migratory and non-migratory behavior, respectively. However, the genomic architecture underlying such behavioral ecotypes is unclear. Here, we have analyzed more than 8.000 polymorphic SNPs distributed throughout all 23 linkage groups and show that loci putatively under selection are localized within three distinct genomic regions, each of several megabases long, covering approximately 4% of the Atlantic cod genome. These regions likely represent genomic inversions. The frequency of these distinct regions differ markedly between the ecotypes, spawning in the vicinity of each other, which contrasts with the low level of divergence in the rest of the genome. The observed patterns strongly suggest that these chromosomal rearrangements are instrumental in local adaptation and separation of Atlantic cod populations, leaving footprints of large genomic regions under selection. Our findings demonstrate the power of using genomic information in further understanding the population dynamics and defining management units in one of the world’s most economically important marine resources.

Genomic differentiation between populations can display complex patterns, involving selection of numerous genome wide loci^1^,^2^,^3^,^4^ and challenge the understanding of the different evolutionary processes that shape such genomic signatures^5^,^6^. The key lies in understanding the genetic architecture of adaptive divergence and the balance between divergent selection and homogenizing gene flow. Genome-wide single nucleotide polymorphism (SNP) analyses as well as large-scale sequencing of natural populations address this challenge by identifying areas of the genome involved in diversification^1^,^2^,^7^,^8^, and sometimes also the underlying candidate genes involved in population divergence^4^,^9^,^10^. In some cases, genomic islands of divergence^5^,^6^ – linked loci within genomic regions under selection – have been observed, whereby elevated levels of divergence between individuals or populations expand over extensive regions^4^,^11^. Such patterns can emerge via divergence hitchhiking^12^ or by other factors that reduce recombination across the genome, such as chromosomal rearrangements and thereby maintaining polymorphism in complex traits^13^. Chromosomal inversion polymorphism may play a key role in the process of local adaptation if it captures several locally adapted alleles since the inversion suppresses meiotic recombination in heterozygous individuals, thereby avoiding the association of adapted/maladapted allele combinations^13^. The identification of a limited number of differentiated genomic regions, in combination with little or no genetic differentiation in other parts of the genome, presumed not to be under selection, is usually interpreted as a sign of ecological differentiation through local adaptation in the compared populations or species^12^,^14^. Indeed, if genetic structuring is low in the presumably neutrally evolving part of the genome, divergent regions are most likely of functional importance^6^,^15^,^16^. Nevertheless, in most species, and in marine species in particular, the potential for such adaptation and the underlying genetic architecture remains unclear.

Atlantic cod is one of the most studied and exploited marine species in the world. Despite this fact, the degree of population structuring^17^ and the potential for local adaptation^18^ remains debated. In 1933, two distinct groups of Atlantic cod were described by Rollefsen^19^, based on growth zones and patterns of otoliths in what is now known as Northeast Arctic cod (NEAC) and Norwegian coastal cod (NCC). Since then, a controversy has existed on whether NEAC and NCC are genetically distinct populations. After more than 80 years of controversy, these issues are still not fully resolved^20^, even though genetic markers under selection, such as hemoglobin^21^ and *Pan* I^22^ display significant frequency differences between NEAC and NCC. The NEAC is characterized by long distance migrations from the spawning grounds along the Norwegian coast to feeding areas in the Barents Sea. The main spawning grounds are off the Lofoten islands^23^ and after spawning, the majority of eggs and larvae drift along the coast into the nursery area in the Barents Sea. In contrast to NEAC, NCC inhabits coastal- and fjord areas along the Norwegian coast, perform relatively short coastal migrations^24^ and spawn along most of the Norwegian coast^25^, including the Lofoten area^26^. To date, it is uncertain if the NCC is a self recruited population or if it is a stock recruited in part also by vagrant NEAC individuals. Several mechanisms have been proposed to hinder the potential for hybridization between NEAC and NCC like lekking spawning behavior^27^, depth/temperature preferences at spawning^26^, drift trajectories of offspring^28^ and settling depth for juveniles^29^. The clearly observed phenotypic diversity between ecotypes of migrating and non-migrating Atlantic cod populations that nonetheless spawn in the vicinity of each other, offers an excellent opportunity to identify the potential for local adaptation and investigate its genomic architecture, when both natural selection and gene flow are potentially high, in a major marine resource.

Population divergence of Atlantic cod populations in northern Norway have so far been described by a small to moderate number of genetic markers^20^,^30^ or by pooled population data^31^, which limits inference of the genomic architecture underlying local adaptation as well as the level of neutral divergence. Nonetheless, distinct regions of elevated divergence have been detected by comparing Atlantic cod populations in other parts of its geographical range^4^,^30^. Moreover, it has been suggested that these regions consist of genomic rearrangements like chromosomal inversions^11^. We here aim at elucidating the genomic distinctions between migratory cod (NEAC) and costal cod (NCC) using the available SNP chip resource featuring more than 8.000 SNPs distributed throughout the genome. An additional comparison with a more remote population from the North Sea enables us to compare the population structure of NEAC and NCC that spawn in the vicinity of each other to a population that spawns at a distinctly different location and hence better quantify the genomic differences within and between different areas. We use population genetic theory and two different outlier approaches to identify SNPs and thus, genomic regions under selection. Distinct genomic regions separating the two populations were demonstrated and chromosomal rearrangement patterns were further investigated, using different approaches based on linkage disequilibrium (LD) and haplotype tagging. Finally, we discuss the mechanisms driving the observed patterns of local adaptation and separation in Atlantic cod and in marine fish species in general.

## Results

**Figure 1:**
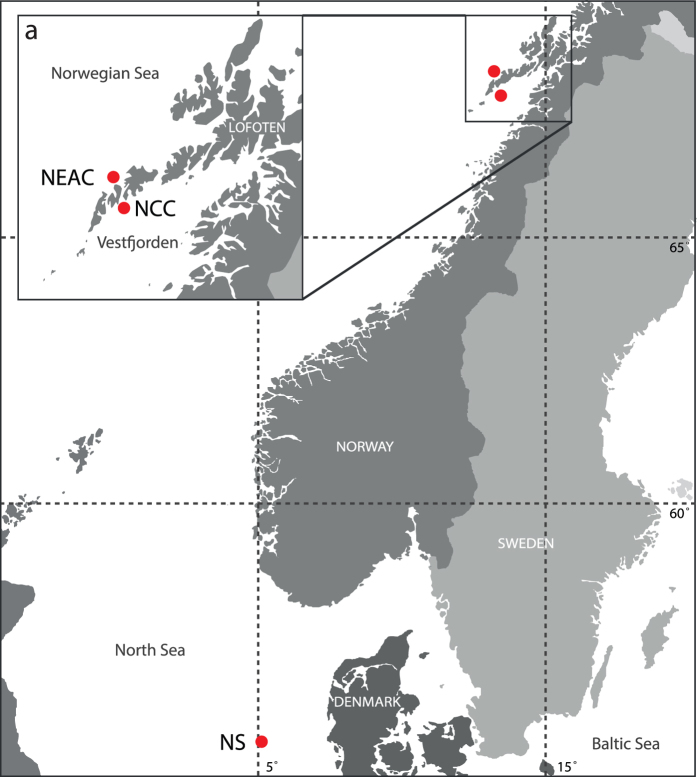
Map of sampling locations for the three Atlantic cod populations used in this study. Red dots indicate the position where the samples were collected. The intersect (**a**) show a detailed view of the Lofoten area. NEAC = Northeast Arctic cod, NCC = Norwegian coastal cod, NS = North Sea cod. See Table 1 for a detailed description of the samples. The map was modified from http://www.graphic-flash-sources.com/europe-free-vector-map/ using Adobe Illustrator CS5.

A total of 8.168 SNPs were analyzed in 141 individuals of Atlantic cod (Fig. 1, Table 1). The SNPs were distributed over all 23 linkage groups^32^,^33^ (LGs) with an average distance between SNPs of 94.000 bp (based on a genome size of 830 Mb)^33^. Out of these SNPs, a total of 5.205 SNPs were located within 5.000 bp of 4.247 Ensembl annotated genes.

### Outlier detection and identification of genomic regions under selection

**Figure 2:**
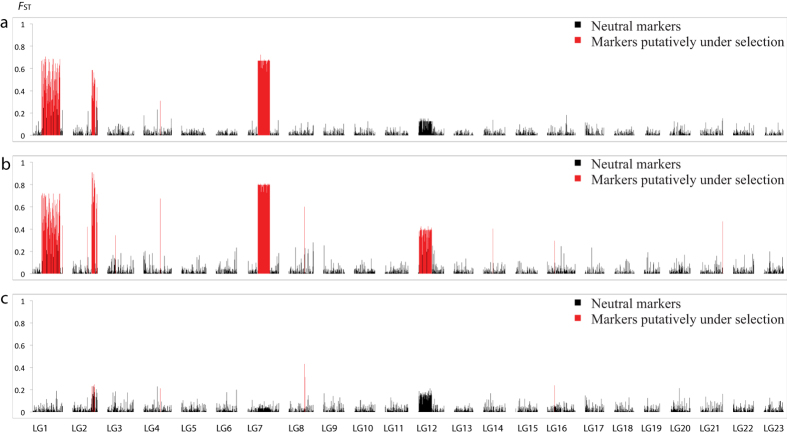
Locus specific *F*_ST_ values for the pairwise population comparisons between Northeast-Arctic, Norwegian coastal and North Sea cod. (**a**) The observed *F*_ST_ pattern indicates three distinct regions of the genome with elevated *F*_ST_ values in the NEAC-NCC comparison. (**b**) In the comparison between NEAC and the geographically more distant NS population, four distinct regions with elevated *F*_ST_ values are observed. (**c**) Only smaller areas of the genome shows elevated *F*_ST_ values in the NCC-NS comparison even though they are collected from geographically distant locations. SNPs are ordered according to linkage group and position within linkage groups. SNPs that are identified as putatively under selection are in red color. NEAC = Northeast Arctic cod, NCC = Norwegian coastal cod, NS = North Sea cod.

The BAYESCAN analyses identified 336 SNPs (4.1%) as candidates for divergent selection in the NEAC/NCC comparison (*q* < 0.01), while FDIST2, implemented in LOSITAN, identified 479 outlier SNPs (5.9%, *q* < 0.01, Supplementary Table S1). All SNPs identified as outliers by BAYESCAN were also identified by LOSITAN, resulting in a final outlier dataset of 336 SNPs (Fig. 2a, Supplementary Table S2). LG1, 2 and 7 have the highest number of outliers: 146, 35, 154 respectively while a single outlier SNP was detected in LG4 (Supplementary Table S2). Out of these SNPs, 244 loci were located in or within 5 Kb of a known gene, of which 134 were located in exons and 114 were non-synonymous substitutions causing amino acid changes. Notably, a single outlier SNP in a gene with unknown function (ENSGMOG 00000011194) was detected in LG4 in the final outlier dataset in addition to a limited number of outlier SNPs in 16 other LGs (Supplementary Table S1) that were only detected as outliers using LOSITAN. These are all single outliers, not representing any larger outlier blocks and not residing within linked regions of the genome, however indicating that a few smaller areas of the genome also could play a role in the genomic diversification of the NEAC and NCC populations. The identified outlier pattern between the NEAC and the NS population (Fig. 2b) resembles the NEAC/NCC comparison, except that outlier signals are generally stronger and 109 additional outlier SNPs were also detected within LG12 (Supplementary Table S1). Nine SNPs were candidates for selection in the comparison between NCC and NS (Fig. 2c, Supplementary Table S1).

SNPs were categorized as outlier SNPs or as neutral SNPs based on the comparison between NEAC and NCC populations. The final outlier dataset of 336 SNPs are represented by 86 tag-SNPs while the neutral dataset of 7.702 SNPs are represented by 7.384 tag-SNPs (details on tag-SNP selection are given in Materials and Methods).

### Population genetic structuring

**Figure 3:**
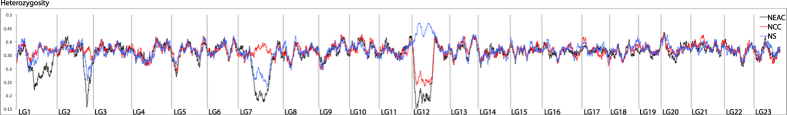
Heterozygosity level across all linkage groups in the Northeast-Arctic, Norwegian coastal and North Sea cod. SNPs are ordered according to linkage group and position within linkage groups. The observed heterozygosity pattern shows four distinct regions of the genome with distinctly different heterozygosity values. NEAC = Northeast Arctic cod, NCC = Norwegian coastal cod, NS = North Sea cod.

*F*_ST_ values of the outlier SNPs (*F*_ST_ = 0.35053) were orders of magnitude larger than those of the neutral SNPs (*F*_ST_ = 0.00123) between the NEAC and NCC populations, and all *F*_ST_ values were significantly different from 0 (Table 2). Moreover, the elevated *F*_ST_ values predominantly occur in LG1, 2 and 7, (Fig. 3a, Supplementary Table S3). We observed slightly larger *F*_ST_ differences when comparing the NEAC population to the more geographically distant NS population (Table 2), predominantly through elevated *F*_ST_ values in LG12 (Fig. 3b, Supplementary Table S3). When comparing the NCC and NS populations, *F*_ST_ values are generally low (Table 2) with small but distinct *F*_ST_ elevations in LG2 and 12 (Fig. 3c, Supplementary Table S3).

**Figure 4:**
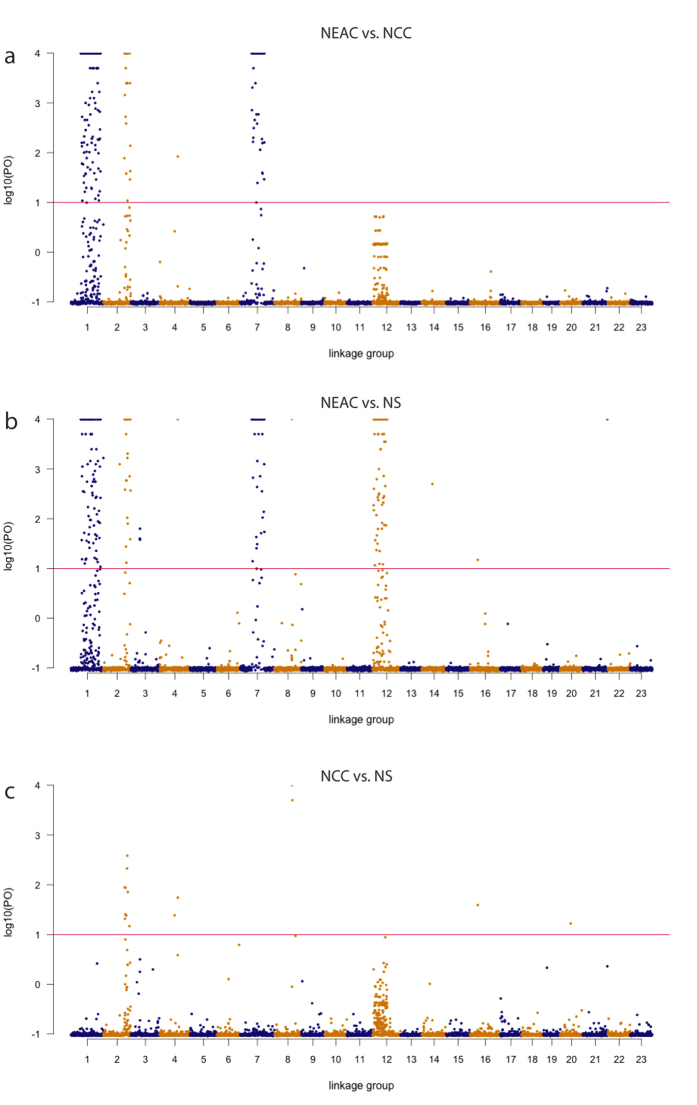
Manhattan plot of pairwise outlier analyses based on median log_10_ (PO) from 10 replicate runs of BAYESCAN. (**a**) The observed outlier pattern between NEAC and NCC indicate that the outliers are clustered within three distinct genomic areas. Only one additional outlier is detected in LG4. (**b**) In the comparison between NEAC and the geographically more distant NS population, an additional outlier area is observed in LG12. (**c**) In the NCC-NS comparison, the outlier area in LG2 is observed, but with lower log_10_ (PO) values. SNPs are plotted according to linkage group and position within the linkage groups along the X-axis. The red line at 1 indicates ‘strong association’ according to Jeffreys^73^. NEAC = Northeast Arctic cod, NCC = Norwegian coastal cod, NS = North Sea cod. For visualization purpose, maximum log10(PO) values are set to 4 and all underlying values are found in Supplementary Table S1.

Based on all SNPs, no private alleles were detected in any of the populations (Supplementary Table S3), although 52 SNPs that were candidates for selection were fixed or close to fixation in the NEAC population (allele frequency >0.95, Supplementary Table S3). Distinctly different patterns of heterozygosity within the different LGs were detected among the populations (Fig. 4, Supplementary Table S3), which correspond well with the areas of high *F*_ST_ values (Fig. 3) as well as the identified outlier regions (Fig. 2). The exact tests for deviation from Hardy-Weinberg equilibrium (HWE) shows that only one SNP was out of HWE (*q* < 0.05; Supplementary Table S3), indicating no presence of a Wahlund effect. The number of polymorphic loci, observed- and expected heterozygosity (*H*_*o*_ and *H*_*e*_) were similar in all populations (Table 1).

Population assignment tests using all 8.168 SNPs correctly assigned 136 of the 141 individuals (96.5%) to their presumed source populations (Supplementary Table S4). The miss assignment is due to one individual in the NEAC sample collection assigning as NCC and four individuals in the NCC sample collection assigning as NEAC. The same assignment pattern is obtained using the neutral SNPs (Supplementary Table S4).

Bayesian cluster analyses as implemented in STRUCTURE support a separation between all three populations using the full dataset and the outlier dataset, while little separation between the NEAC and the NCC populations was detected using the neutral dataset (Supplementary Fig. S1). The DAPC analysis, using all SNPs, confirms this population structure (Supplementary Fig. S2a). Moreover, the structure in these data is driven by the three regions within LG1, 2 and 7, according to the DAPC loading plots (Supplementary Fig. S2b).

### Linkage disequilibrium patterns and chromosomal rearrangements

**Figure 5:**
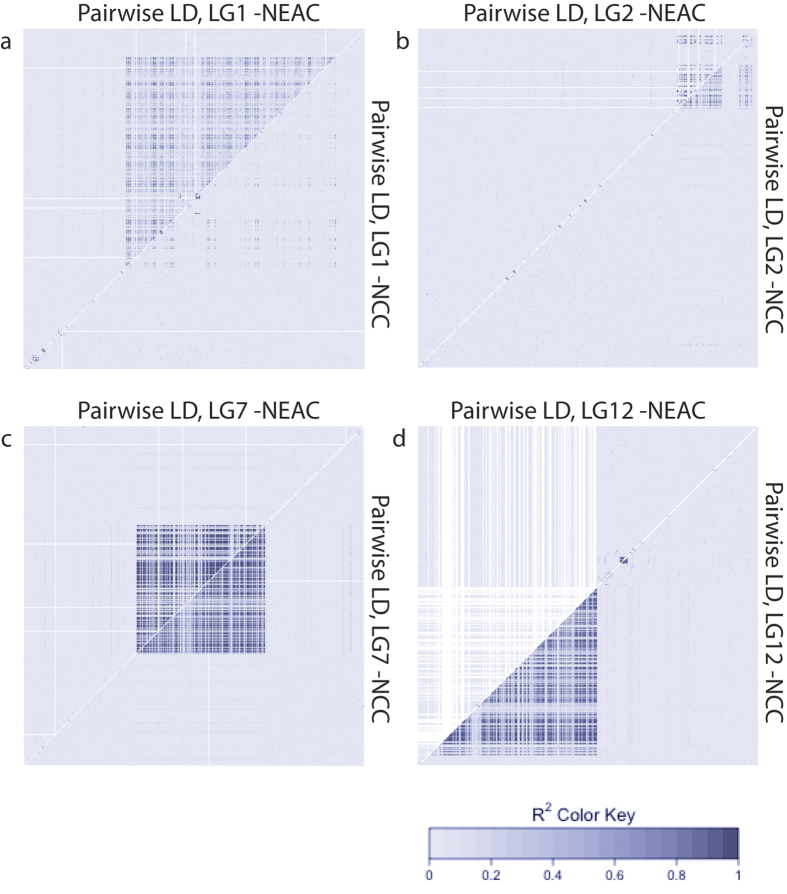
Linkage disequilibrium in linkage group 1, 2, 7 and 12. Pair-wise LD among loci, measured as *r*^*2*^, are estimated within the Northeast-Arctic cod (above the diagonal) and Norwegian coastal cod (below the diagonal) populations. (**a, d**) A distinctly different LD pattern is observed between NEAC and NCC in LG1 and 12. (**b, c**) The LD pattern is similar within LG2 and 7 between NEAC and NCC. Corresponding patterns for all other linkage groups and for North Sea cod are shown in Supplementary Fig. S3 while the underlying LD measurements are found in Supplementary Table S5. NEAC = Northeast Arctic cod, NCC = Norwegian coastal cod.

**Figure 6:**
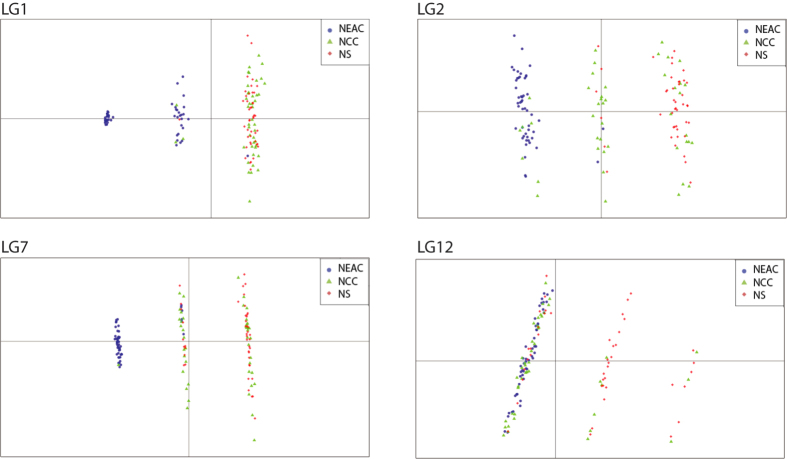
The population structuring within the rearranged regions in LG1, 2, 7 and 12 in Atlantic cod. The first two prinsipal components obtained from PCA of the NEAC, NCC and NS populations, using markers within the rearranged regions in the respective LGs. Each dot represents an individual and the left and right hand clusters represents the homozygotes while the middle cluster represents the heterozygotes. Corresponding patterns for all other linkage groups are shown in Supplementary Fig. S5. NEAC = Northeast Arctic cod, NCC = Norwegian coastal cod, NS = North Sea cod.

A substantial number of SNPs in high LD are detected within LG1, 2, 7 and 12 (Fig. 5a–d, Supplementary Table S5) and the LD pattern is distinctly different between the NEAC and the two other populations in LG1 and 12 (Fig. 5a,d, Supplementary Fig. S3). The LD analyses also reveal eight smaller regions of high LD (Table 3, Supplementary Fig. S3). By using the *R* package inveRsion, the linked regions in LG1, 2, 7 and 12 were suggested as inversions (Supplementary Fig. S4) and the identified breakpoints correspond well with the identified boundaries for the blocks in high LD (Table 3). Different genotypic combinations of the linked alleles at LG1, 2, 7 and 12, contribute to the observed population divergence (Table 3, Fig. 6), which support the hypothesis that these regions are chromosomal rearrangements. By defining the identified LD areas as regions of interest in the InvClust package, also the smaller regions of high LD are suggested as inversions (Supplementary Fig. S4) but they do not show a population based allele distribution between NEAC and NCC (Table 3, Supplementary Fig. S5).

The identified LD block on LG1 covers at least 18.5 Mb containing 785 genes (Supplementary Table S6) and shows a distinctly different LD pattern in the NEAC and NCC populations (Fig. 5a). Two smaller and separate LD blocks towards the end of LG2 were identified that cover approximately 5 Mb containing 189 genes (Supplementary Table S6). The identified LD block on LG7 covers at least 9.5 Mb and 297 genes (Supplementary Table S6). In these two latter LGs, the LD pattern is similar in both populations (Fig. 5b,c). The SNPs under selection in all of these there LGs, fall within the identified regions of high LD. Combined, the outlier regions in LG1, 2 and 7 cover more than 33Mb (≈4% of the genome) and contain more than 1.200 genes (Supplementary Table S6). In LG12, we observe a pattern where the identified LD block covers at least 12.5 Mb and the LD pattern is distinctly different in the NEAC population relative to the other two populations (Fig. 5d). Whereas, no significant outliers between NEAC and NCC were detected in LG12, outliers spanning the entire linked region were detected between NEAC and the physically more distant NS population.

Relatively high values of LD (*r*^*2*^ > 0.3) occur inter-chromosomally between SNPs on different LGs (Supplementary Table S5). Notably, the outlier SNPs in the LD blocks in LG1, 2 and 7 has *r*^*2*^ values between 0.3 and 0.65 (Supplementary Fig. S6), and the linkage disequilibrium between the divergent blocks in LG1 and LG7 is significant (Fishers' exact test; *p* = 0.0199).

## Discussion

Here we provide new insight into the evolution of distinct ecotypes of Atlantic cod with different life history strategies. We identify a set of three divergent regions between NEAC and NCC that combined span approximately 4% of the genome. The sizes of these regions, in combination with strong linkage patterns, distinct *F*_ST_ pattern and a population specific distribution, suggest that these genomic islands of divergence are the results of chromosomal rearrangements. This divergence is in contrast to the remaining parts of the genome, which is characterized by low levels of genomic divergence. Overall, the data indicate a key role for several chromosomal rearrangements in protecting adaptive loci from recombination^13^ and hence facilitating adaptive genomic divergence.

Genomic islands of divergence have previously been reported in Atlantic cod^4^,^30^,^34^,^35^,^36^ and have been discussed in the context of divergence hitchhiking^4^,^35^. Genomic islands of divergence can also be caused by factors that reduce recombination across the genome such as chromosomal rearrangements where distinct LD blocks will contain the entire rearrangement. Chromosomal rearrangements reduce the rate of crossing over by several orders of magnitudes^37^ and may affect large genomic areas^38^,^39^. As a result, chromosomal rearrangements allow genomic islands of divergence to be larger than in collinear regions^14^,^40^. Our observations of three distinct and large genomic islands in the NEAC/NCC comparison and the additional large island in the NEAC/NS comparison are in high LD with each other throughout the entire block (Supplementary Table S5). This, in combination with population-specific distribution of the haplotype blocks, suggests that these regions are chromosomal rearrangements (Table 3), possibly large inversions, containing outlier SNPs nested within the regions. A plausible explanation for the divergence in the rearranged loci between the ecotypes, considering the low level of divergence in the remaining parts of the genome is that loci within the rearrangements are indicative of strong adaptive divergence. This is in line with Sodeland *et al*.^11^, where rearrangements in LG2, 7 and 12 are identified within coastal and offshore samples of Atlantic cod on the Norwegian Skagerrak coast. Given that the observed rearrangements are inversions, sets of genes involved in local adaptation are either captured and protected from recombination within the inversion^13^ or the position of the inversion breakpoints are giving an evolutionary advantage by changing reading frames or expression patterns^41^. To discriminate between these two explanations require further studies of the inversion breakpoints in multiple populations, using either a much denser SNP chip or a whole genome sequencing approach. Either way, the haplotypes detected here, likely provide selective advantage and are protected by the inversion. The fact that, even though there are population specific variation, all inversion haplotypes are in Hardy-Weinberg equilibrium with no heterozygote deficiencies indicate that there are no genomic disadvantages associated with the heterozygote variant.

Reduced recombination rates at chromosomal centromeres could also explain increased differentiation in localized parts of the genome^42^, but does not explain the observed difference in genomic divergence within these regions (Fig. 3, Table 3). An alternative explanation for the genomic islands of divergence is divergence hitchhiking. In such a scenario, LD is expected to gradually decrease with distance from the target of selection^43^ and as a consequence of recombination events, islands of divergence are expected to be relatively small. Large islands of divergence, as the ones observed here, can potentially be observed if there are several targets of selection within the genomic island^12^,^44^ in addition to sequential buildup of divergence around the targets of selection^45^. However, as LD seems to be persistently high throughout the genomic islands of divergence, this seems to be a less plausible explanation for our results.

The divergent and potentially inverted region in LG1 (Fig. 2a) shows distinct population based frequency distribution (Table 3, Fig. 6). The region covers at least 31 cM according to the linkage map by Hubert *et al*.^32^ and corresponds well with the 23 cM genomic region associated with a migratory ecotype, defined by Hemmer-Hansen *et al*.^30^. Interestingly, this region was not detected as significantly divergent between eastern and western Atlantic cod populations by Bradbury *et al*.^36^, indicating that the selecting agents on this genomic area may be similar on both sides of the Atlantic Ocean or that the inversion event happened before the split between East- and West Atlantic cod populations, approximately 100.000 years ago^46^. Further research on the inversion status of West Atlantic cod samples is needed to unravel this phenomenon. However, the divergent area is tightly linked (Fig. 5a) and displays low heterozygosity (Fig. 3) and low nucleotide diversity (Table 3) in the NEAC population but not so in the NCC population, suggesting that the NEAC population is dominated by an inversion haplotype of a more recent origin than the other two populations, which presumably are dominated by a collinear haplotype (Table 3). Alternatively, a scenario of recent selective sweep may have caused the reduced heterozygosity and nucleotide diversity within the inversion haplotype in the NEAC population. In LG2 and 7, the divergent regions, which also shows distinct population differentiation (Table 3, Fig. 6), coincide with the regions detected by Bradbury *et al*.^36^ and Hemmer-Hansen *et al*.^30^ on both sides of the Atlantic Ocean and by Berg *et al*.^4^ in the Baltic Sea. These regions have been associated with variation in temperature^36^ and/or salinity and oxygen level^4^. The fact that similar regions are found to be divergent in a wide range of populations across the Atlantic Ocean, indicates that these regions could be predating the split between West- and East Atlantic cod populations and that they potentially play a role in local adaptation in multiple ecological settings. Interestingly, balancing selection has recently been described in the *Ckma* gene^47^, which is nested within the divergent region in LG7. The authors suggest that selection could be acting on a larger part of the genome that is locked together by structural variation, which is consistent with our observations. In LG12, the identified divergent region that has been shown to be associated with temperature in two previous studies^4^,^36^, are present on both sides of the Atlantic Ocean and have recently been used to discriminate between fjord and coastal samples of Atlantic cod on the Norwegian Skagerrak coast^11^. Even though this divergent region differentiates between all investigated populations (Table 3, Fig. 6), it is not identified as an outlier region in the NEAC/NCC comparison (Fig. 2) and is likely not responsible for the differentiation between migrating and non-migrating ecotypes *per se*. Interestingly, low heterozygosity was detected in both NEAC and NCC (Fig. 3) but low nucleotide diversity (Table 3) was only detected in NEAC. At the same time, increased heterozygosity and nucleotide diversity was observed in the NS population (Fig. 3, Table 3) indicating that the NEAC and NCC variant could be derived from the NS variant as it only captures a fraction of the genetic variability. In addition to the rearrangements in LG1, 2, 7 and 12, smaller regions of high LD were also suggested as rearrangements in other parts of the genome (Table 3, Supplementary Fig. S5). Although these areas are not under selection and do not contribute to population divergence between NEAC and NCC, their presence shows that such rearrangements can persist in the genome (and may be targets of selection in other populations). Moreover, the detection of these regions shows that it is not the selection *per se* that allows their identification, but rather their specific genomic properties.

As both neutral and selective forces shape the genetic makeup among populations, it is important to disentangle these effects. Even though the majority of the investigated SNPs shows low levels of genetic differentiation (Fig. 3a), large *F*_ST_ differences (Table 2) and genomic regions under selection (Fig. 2a) are observed between the NEAC and the NCC populations, suggesting adaptive genomic divergence. *F*_ST_ values based on the unlinked neutral SNP set indicate that there is a low but significant neutral genetic differentiation between NEAC and NCC (Table 2), confirming that these are truly biological distinct populations. The finding of significant neutral divergence between NEAC and NCC supports the ‘historical isolation hypothesis’ rather then the ‘divergent selection hypothesis’ (both described in^20^), where historic differentiation between NEAC and NCC, presumably in allopatry, allows for these two populations to occupy the same spawning habitats without interbreeding.

Importantly, the well-studied pantophysin gene (*Pan* I) which have been used to determine individuals as either stationary or migratory^22^,^48^ is only one out of approximately 785 target genes in the outlier region on LG1, but could be used as a proxy for the region under selection as a whole. The estimated frequency, based on the inversion status of the entire divergent region (Table 3), corresponds well with the allele frequency of the investigated *Pan* I locus (Supplementary Table S1) and are somewhat higher than, but still in line with frequencies of the *Pan* I locus in the initial study by Fevolden and Pogson^22^. Since this divergent area in LG1 appears to be inherited as one large rearranged region, it is not necessarily indicating that the *Pan* I locus is under selection. Other genes within the same region have also been described as likely targets of selection, such as rhodopsin^49^ and further research is needed to unravel the actual targets of selection.

A natural next step to unravel the history of these and potentially other chromosomal rearrangements is to investigate if the exact locations of inversion breakpoints are shared across a diverse group of Atlantic cod populations. Such analyses would require dense SNP coverage or preferably a whole genome sequencing approach. Our study corroborate by and extent previous work on marine populations, where natural selection shapes the population structure on short spatial scales, despite the high dispersal capacity of these marine organisms^50^,^51^,^52^,^53^. The findings reported here show that the majority of the Atlantic cod genome shows little genetic differentiation between NEAC and NCC, apart from 3 distinct genomic regions that are likely the results of chromosomal inversions, maintained under strong diversifying selection. Hence, any linked genetic markers within the respective areas could be used as proxies for the inverted regions as a whole in population based studies.

## Materials and Methods

### Sample collection, DNA extraction and genotyping

We collected 99 Atlantic cod specimens near the Lofoten Islands at different sampling times (Table 1). Samples collected during spawning time at spawning grounds are here defined as the NEAC population that migrate from the Barents Sea to spawn in the Lofoten area. The samples collected in late June/early July after the spawning NEAC has left the area, are here defined as the resident NCC population. For comparative reasons, a physically more distant population, spawning in a different area, consisting of 42 individuals from the North Sea was included (Fig. 1, Table 1). All fish samples in this study were harvested for human consumption, from which small tissue samples were collected (post mortem). Sampling in this manner does not fall under any specific legislation in Norway, and is in accordance with the guidelines set by the ‘Norwegian consensus platform for replacement, reduction and refinement of animal experiments’ (www.norecopa.no).

DNA was extracted from ethanol stored muscle tissue, using the E.Z.N.A Tissue DNA kit (Omega Bio-Tek, Norcross, GA, USA) and normalized to 100 ng/μl measured on a NanoDrop DN1000 instrument (Thermo Scientific Inc.), accepting only DNA extractions with a 260/280 ratio >1.8 and a 260/230 ratio >2.0. All individual samples were genotyped using a 12 K Illumina SNP-chip. The 12 K SNP-chip was designed, based on re-sequencing and alignment to the Atlantic cod reference genome^33^, of 8 globally collected individuals where all three populations in this study were represented. Out of the 10.913 SNPs on the final SNP-chip, 8.164 SNPs were polymorphic in the NEAC/NCC populations, had a call rate >95% and showed Mendelian inheritance in a set of >2000 family individuals (data not shown). Genotype clustering and pedigree check was performed using the Genome Studio 2011.1 software from Illumina, where each individual SNP locus was manually inspected and clusters were adjusted if appropriate. In addition, 4 SNPs (one hemoglobin SNP, two Rhodopsin SNPs and one *Pan* I SNP) were genotyped on the MassARRAY (Sequenom Inc.), resulting in a final number of 8.168 SNPs with a minor allele frequency (MAF) >0.05 in any population. Out of these, 602 SNPs were close to selected candidate genes, 1.470 SNPs were non-synonymous coding SNPs while the remaining 5.857 were SNPs randomly distributed throughout the 23 different LGs (the source and selection criteria of each SNP is listed in Supplementary Table S1). The nomenclature of LGs in this paper follows Hubert *et al*.^32^ while the order of the SNPs are based on preliminary linkage data as in Berg *et al*.^4^.

### Population genetics, linkage disequilibrium and rearrangement patterns

Within each population, estimates of observed (*H*_*o*_) and expected heterozygosity (*H*_*e*_) were calculated in ARLEQUIN 3.5.1.3^54^. Departure from Hardy-Weinberg Equilibrium (HWE) was tested locus by locus in each population using the exact test in ARLEQUIN with 100.000 iterations and a Markov Chain of 1.000.000. Correction for multiple testing were done by computing the *q*-value for each locus, using a *q*-value of 0.05 as a threshold for significance, using the QVALUE package^55^ in *R*^56^. Allele frequencies were calculated for all SNPs in all three populations using ARLEQUIN.

SNPs were categorized as outliers or as neutral, based on the outlier analyses in the NEAC and the NCC material (see next section). To avoid bias in the datasets, neutral and outlier SNPs in LD with each other (*r*^*2*^ > 0.5) were represented by tag SNPs, selected using PLINK v1.07^57^ and used in the *F*_ST_ and STRUCTURE analyses. Locus specific *F*_ST_ values and weighted average *F*_ST_ values between the three populations were calculated for the full-, the neutral- and the outlier datasets, using ARLEQUIN. For all comparisons, 10.000 permutations were used. We calculated the nucleotide diversity (π) within all populations and in all populations combined, using a sliding windows approach with a 50-SNP window and 10 SNPs per iteration in DnaSP 5.10^58^.

We used the program STRUCTURE v2.3.4^59^ to identify major genetic clusters in the dataset, using the correlated allele frequency and admixture model to best reflect the most likely pattern of population connectivity. We performed 10 independent runs for each value of *K*, with *K* = 1–4 (burn in of 10.000 MCMC iterations followed by 100.000 MCMC iterations). Visualization and evaluation of the best *K*-value for the individual STRUCTURE runs was performed, using CLUMPAK^60^. We performed discriminant analysis of principal components (DAPC, using all 8.168 SNPs in the *R* package ADEGENET^61^. DAPC is a method that relies on data transformation using principal component analysis (PCA) prior to the discriminant analysis (DA) step, ensuring that variables in the DA analysis are uncorrelated. Further, loadingplots from DAPC was used to identify the main SNPs that are driving the genetic divergence among the three populations.

We evaluated the presence of linkage disequilibrium (LD) in all three populations separately using all 8.168 SNPs, reporting both inter- and intra- chromosomal LD, quantified with the *r*^2^ estimate, using PLINK. Different approaches were used to investigate rearrangement patterns in the data. We used the *R* package inveRsion^62^, which is based on LD differences across inversion breakpoint, to detect and locate large inverted genomic regions and to identify the inversion status of each individual, using block size = 3, min. allele = 0.1 and thbic = 0. By defining regions of interest, based on the LD analyses, we also used the *R* package invClust^63^, which is based on haplotype tagging and dimensionality reduction analysis, to assess if the identified LD regions in the Atlantic cod genome were likely to be inversions, visualized as a three-cluster pattern in the first component in a multidimensional scaling (MDS) analysis. This method was also used to identify the inversion status of all individuals within each inverted region. Finally, a method described by Ma^64^, where PCA is performed locally within the identified inversions, was used to confirm and visualize the distribution of individuals to their inversion status, based on their population of origin.

### Assignment testing and outlier detection

Assignment of all individuals to their presumed source populations was estimated using the Bayesian assignment method in GENECLASS2^65^, employing the ‘leave-one-out’ procedure. Based on the assignment tests, using all 8.168 SNP markers, a dataset for the outlier analyses was defined. This dataset contains only individuals from the source populations of NEAC and NCC (where 5 miss-assigned individuals were excluded) in addition to 42 NS samples, hence containing 136 individuals.

Two independent methods were used to identify candidate loci under selection in all population pairs. First, we used a Bayesian regression approach implemented in BAYESCAN v2.1^66^ which, measures the discordance between global and population-specific allele frequencies, based on *F*_ST_ coefficients. To control for variation in the Bayes Factor (BF) distribution caused by randomness in each run of BAYESCAN, the median value of 10 independent runs were calculated for each SNP. We carried out the simulations, using stringent criteria, assuming selection to be 10%. The false discovery rate (FDR) was set to 0.01. We report both the median log_10_ value of the posterior odds (PO) as well as the *q*-value for each SNP in all pairwise comparisons. Second, we used the FDIST2 approach implemented in the software LOSITAN^67^, where comparisons are made of *F*_ST_ values in relation to heterozygosity of individual loci, based on a neutral distribution. We carried out the simulations, under the Infinite Allele Method (IAM) with 1.000.000 simulations, a confidence interval of 0.99 with a FDR of 0.01; using the “neutral” mean *F*_ST_ option and forcing mean *F*_ST_ option. Based on the probabilities calculated in LOSITAN, *q*-values were calculated and reported for all pairwise comparisons, using the QVALUE package in *R* (using a *q*-value of 0.01 as a threshold for significance). To corroborate our choice of cutoff value, and to be able to use a consistent cutoff value in both the BAYESCAN and the LOSITAN analyses, we used both *q*-values AND cutoff values based on Log_10_ (PO) in determining the significance level in both outlier tests. The consistency of the different approaches for outlier detection and the strength of the identified outlier loci, strongly suggest that the majority of the identified loci and their associated genomic regions are subject to divergent selection. Nevertheless, some outlier loci are only detected by a single approach. This variation can either be caused by different underlying assumptions and detection rates of BAYESCAN and LOSITAN or slightly different cutoff criteria used^68^. It has been suggested that outlier tests may have high false positive rates due to the effects of population demography and bottleneck effect^69^. Even though this is less likely in our case due to presumably large population sizes and shallow neutral population structuring, we performed outlier analyses between pairs of populations, partly omitting the methodological weakness of population structure in the datasets^70^.

### SNP annotation

All 8.168 SNPs used, were mapped to the published Atlantic cod genome (ATLCOD1C)^33^, for which Ensembl annotation is available, in the same way as in Berg *et al*.^4^. SNPs located either within a gene or located within a 5000 bp region up or downstream of a gene were identified using BEDclosest^71^ with the option -t “first” and -d to determine distance. Further, protein transcripts of Ensembl genes that were associated with the location of the SNPs through this approach were annotated with BLAST2GO^72^ using public database b2g_sep13. Protein transcripts were aligned to the refseq_protein data using the BlastP algorithm in BLAST2GO, allowing a maximum of 20 hits with a minimum e-value of 1E-3. Apart from setting the evidence code weight of IEA (electronic annotation evidence) to 1, default weights were used. Annotation was augmented using the Annex function in BLAST2GO. All SNPs are referred to by their ss# or rs# available in dbSNP (www.ncbi.nlm.nih.gov/SNP/). All raw data are provided in Supplementary File S1 in PLINK format, where the ped file contains all genotypes for all individuals and all 8168 SNP markers and the map file contains all SNP names.

## Additional Information

**How to cite this article**: Berg, P. R. *et al*. Three chromosomal rearrangements promote genomic divergence between migratory and stationary ecotypes of Atlantic cod. *Sci. Rep.* **6**, 23246; doi: 10.1038/srep23246 (2016).

## Supplementary Material

Supplementary Information

Supplementary Information

Supplementary Information

Supplementary Information

## Figures and Tables

**Table 1:** Atlantic cod samples included in this study and basic population genetic parameters.

Sampling ID	Sampling time	Lat.	Long.	Condition	Sample size	Ind. call no. >0.95	Avg. call rate	# Poly-morphic loci	*Ho* (s.d.)	*He* (s.d.)
Northeast Arctic cod (NEAC)	Mar 2011	N68.19	E13.30	Adults, spawn.	51	51	0.983	8113	0.354 (0.149)	0.353 (0.139)
Norwegian coastal cod (NCC)	Jun/Jul 2011	N68.04	E13.41	Adults/juv.	48	48	0.995	8121	0.364 (0.140)	0.369 (0.128)
North Sea cod (NS)	Mar 2002	N55.60	E05.85	Adults, spawn.	42	42	0.982	7953	0.367 (0.144)	0.365 (0.133)

Estimates of observed (*H*_*o*_) and expected heterozygosity (*H*_*e*_) were calculated using ARLEQUIN^54^.

s.d. = standard deviation, Latitude and longitude values are given in degrees and minutes.

For sample details on each of the individuals, see Supplementary Table S4.

**Table 2:** Pairwise *F*_ST_ values among Atlantic cod populations, using full-, neutral- and outlier datasets.

*F*-statistics			
	Full dataset(8.168 SNPs)	Neutral dataset(7.384 tag-SNPs)	Outlier dataset(86 tag-SNPs)
NEAC/ NCC	0.04246	0.00123	0.35053
NEAC/ NS	0.06237	0.00861	0.37502
NCC/ NS	0.00839	0.00519	0.01410

NEAC = Northeast Arctic cod.

NCC = Norwegian coastal cod (Lofoten).

NS = North Sea cod.

All *F*_ST_ values are significant values (*p*-values < 0.0000.

10.000 permutations used) calculated in ARLEQUIN^54^.

**Table 3:** Chromosomal rearrangements in Atlantic cod, their contribution to population genetic structure and nucleotide diversity within these regions.

LG	LD area*(SNP no.)	Rearrangement frequencies									Genotypic differentiation			Nucleotide diversity				Identified breakpoint
		NEAC			NCC			NS			NEAC/NCC	NEAC/NS	NCC/NS	(π)				
		AA	AB	BB	AA	AB	BB	AA	AB	BB				NEAC	NCC	NS	All	
1	137–417	0.02	0.49	0.49	0.85	0.17	0.00	0.98	0.02	0.00	**0.000**	**0.000**	0.139	0.24	0.35	0.33	0.39	134–417
2	751–837	0.96	0.04	0.00	0.19	0.44	0.37	0.00	0.14	0.86	**0.000**	**0.000**	**0.000**	0.21	0.36	0.25	0.37	749–835
4	1391–1413	0.16	0.51	0.33	0.29	0.50	0.21	0.36	0.50	0.14	0.170	0.012	0.626	0.31	0.31	0.26	0.28	
7	2539–2719	0.00	0.14	0.86	0.50	0.42	0.08	0.75	0.25	0.00	**0.000**	**0.000**	0.022	0.19	0.34	0.22	0.43	2537–2720
10	3660–3690	0.27	0.51	0.22	0.23	0.56	0.21	0.30	0.43	0.27	0.965	0.837	1.000	0.41	0.40	0.41	0.39	
11	3890–3909	0.49	0.37	0.14	0.33	0.48	0.19	0.43	0.32	0.25	0.246	0.359	1.000	0.40	0.40	0.38	0.39	
12	4250–4462	0.98	0.02	0.00	0.77	0.17	0.06	0.36	0.48	0.16	**0.004**	**0.000**	**0.003**	0.17	0.31	0.43	0.34	4248–4444
17	6047–6069	0.63	0.33	0.04	0.37	0.46	0.17	0.70	0.25	0.05	0.015	0.667	**0.007**	0.40	0.43	0.39	0.41	
19	6639–6650	0.53	0.29	0.18	0.46	0.37	0.17	0.25	0.55	0.20	0.920	0.095	0.213	0.39	0.39	0.41	0.40	
20	6877–6890	0.63	0.35	0.02	0.50	0.40	0.10	0.45	0.48	0.07	0.190	0.112	1.000	0.39	0.39	0.35	0.34	
21	7203–7222	0.29	0.55	0.16	0.33	0.46	0.21	0.39	0.36	0.25	1.000	0.864	1.000	0.38	0.41	0.40	0.39	
23	7991–8005	0.43	0.45	0.12	0.46	0.44	0.10	0.48	0.43	0.09	0.920	0.667	1.000	0.41	0.42	0.39	0.40	

NEAC = Northeast Arctic cod, NCC = Norwegian coastal cod, NS = North Sea cod. AA = frequency of the least common rearrangement variant in the total material, BB = frequency of the most common rearrangement variant in the total material. All rearrangements are detected with InvClust^62^. Rearrangements in LG1, 2, 7 and 12 are also detected with InveRsion^63^. Identified breakpoints are from InveRsion, where left (min) and right (max) values are used. As InvClust does not detect breakpoints, values are missing for rearrangements that are only identified with InvClust. Genotypic differentiation is given in *q*-values. Significant values (*q* < 0.01) are written in bold text. Nucleotide diversity (π) is calculated in DnaSP^58^. MDS plots for all rearrangements are given in Supplementary Fig. S4.

^*^Identified LD area covering more than 10 SNPs. SNP no. corresponds to the SNP order in Supplementary Table S1.
