# Supplementary material for: Three chromosomal rearrangements promote genomic divergence between migratory and stationary ecotypes of Atlantic cod
Source: Sci Rep. 2016 Mar 17;6:23246. doi: 10.1038/srep23246 (PMC4794648; doi:10.1038/srep23246)
Supplement: Supplementary Information [file srep23246-s2.pdf]

Three chromosomal rearrangements promote genomic divergence between migratory and stationary ecotypes of Atlantic cod

Paul R. Berg, Bastiaan Star, Christophe Pampoulie, Marte Sodeland, Julia M. I. Barth, Halvor Knutsen, Kjetill S. Jakobsen and Sissel Jentoft.

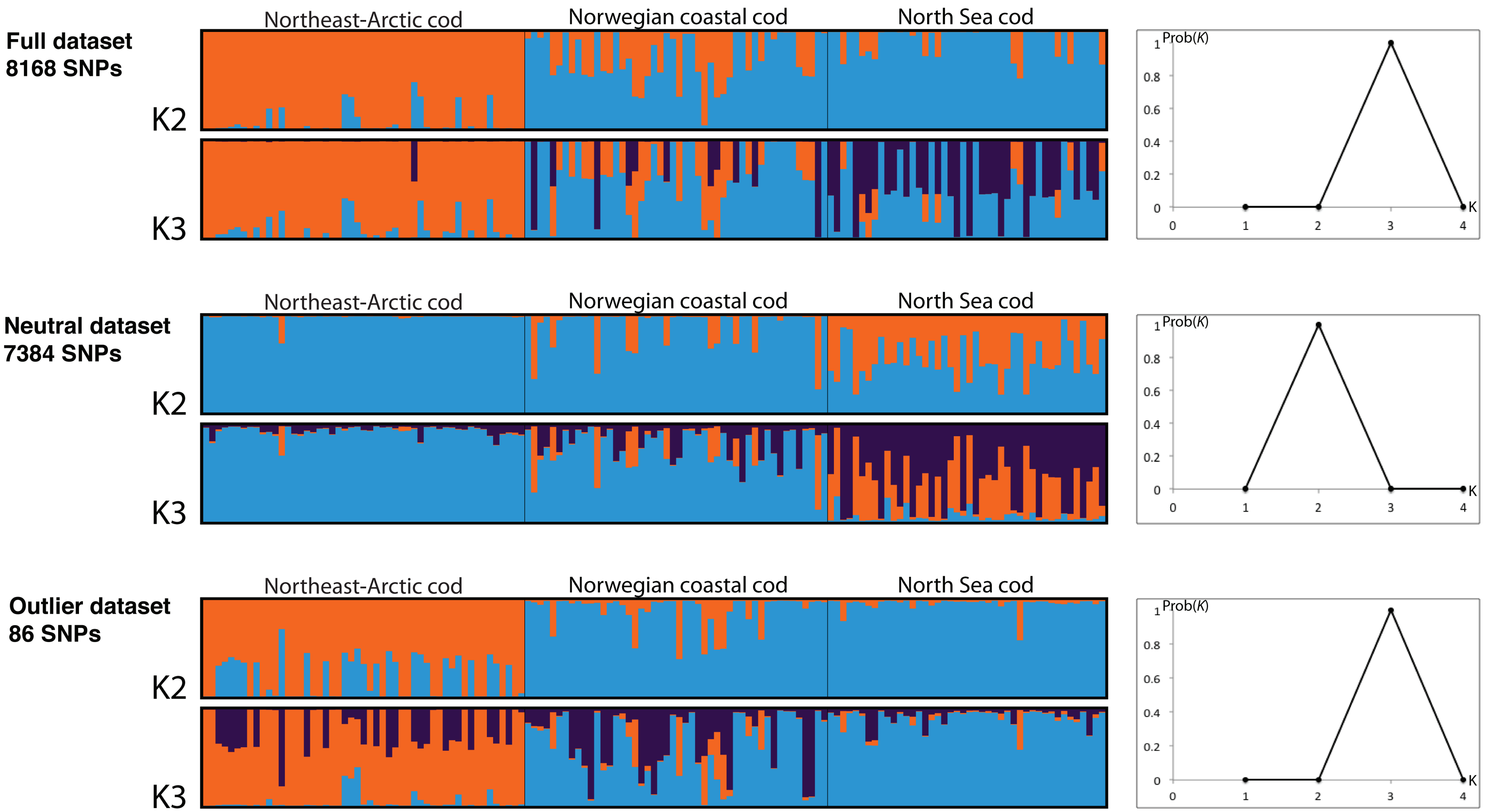

Supplementary Figure S1. **Structure plots of the assignment probabilities in Northeast-Arctic cod, Norwegian coastal cod and North Sea cod.** A bar represents each individual sample. The plots are presented for the full-, the neutral- and the outlier datasets, based on the combined results from 10 independent STRUCTURE runs, for *K* values 2 and 3. The probabilities for the most likely *K* value are shown to the right.

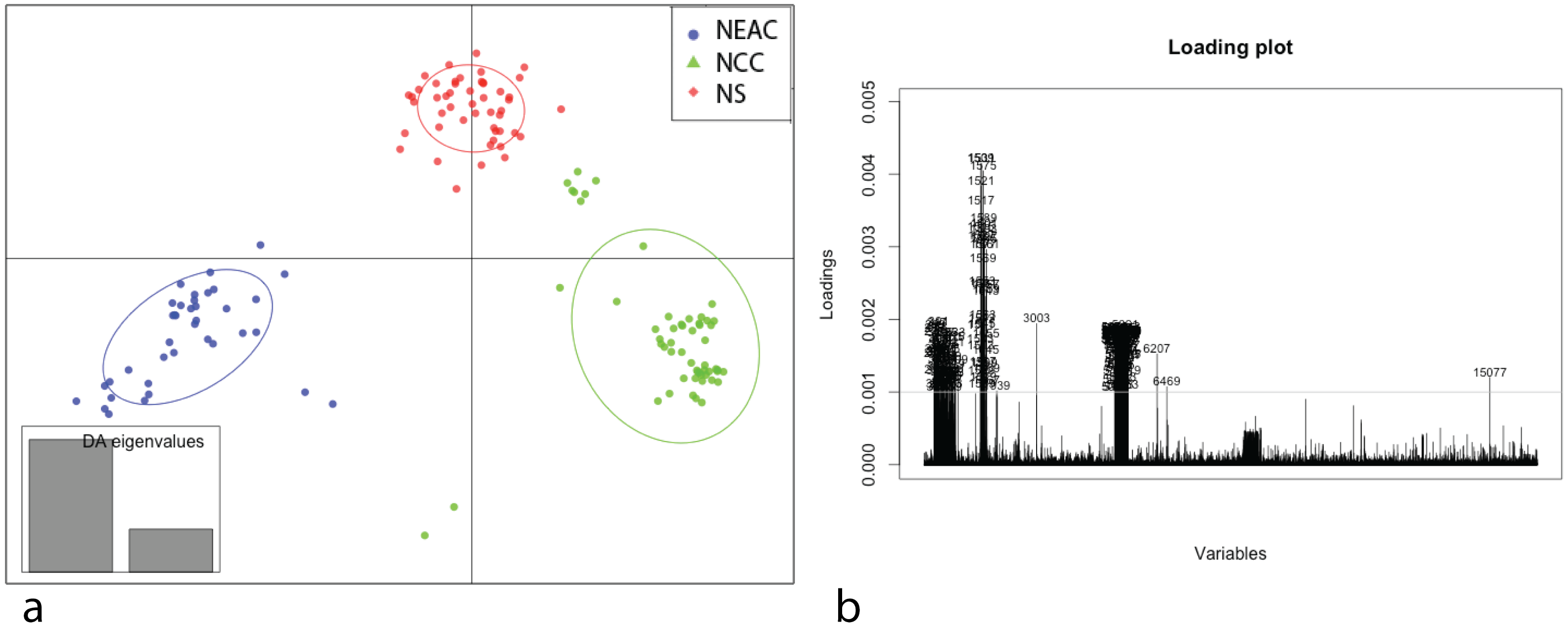

Supplementary Figure S2. **(a) Discriminant analysis principal component (DAPC) analysis showing the spatial relationship between Northeast-Arctic cod, Norwegian coastal cod and North Sea cod.** DA eigenvalues are shown in the left hand corner. **(b) Loading plot based on DAPC analysis.** The plot shows the contribution of each SNP to the differentiation between the Northeast-Arctic cod, Norwegian coastal cod and North Sea cod populations.

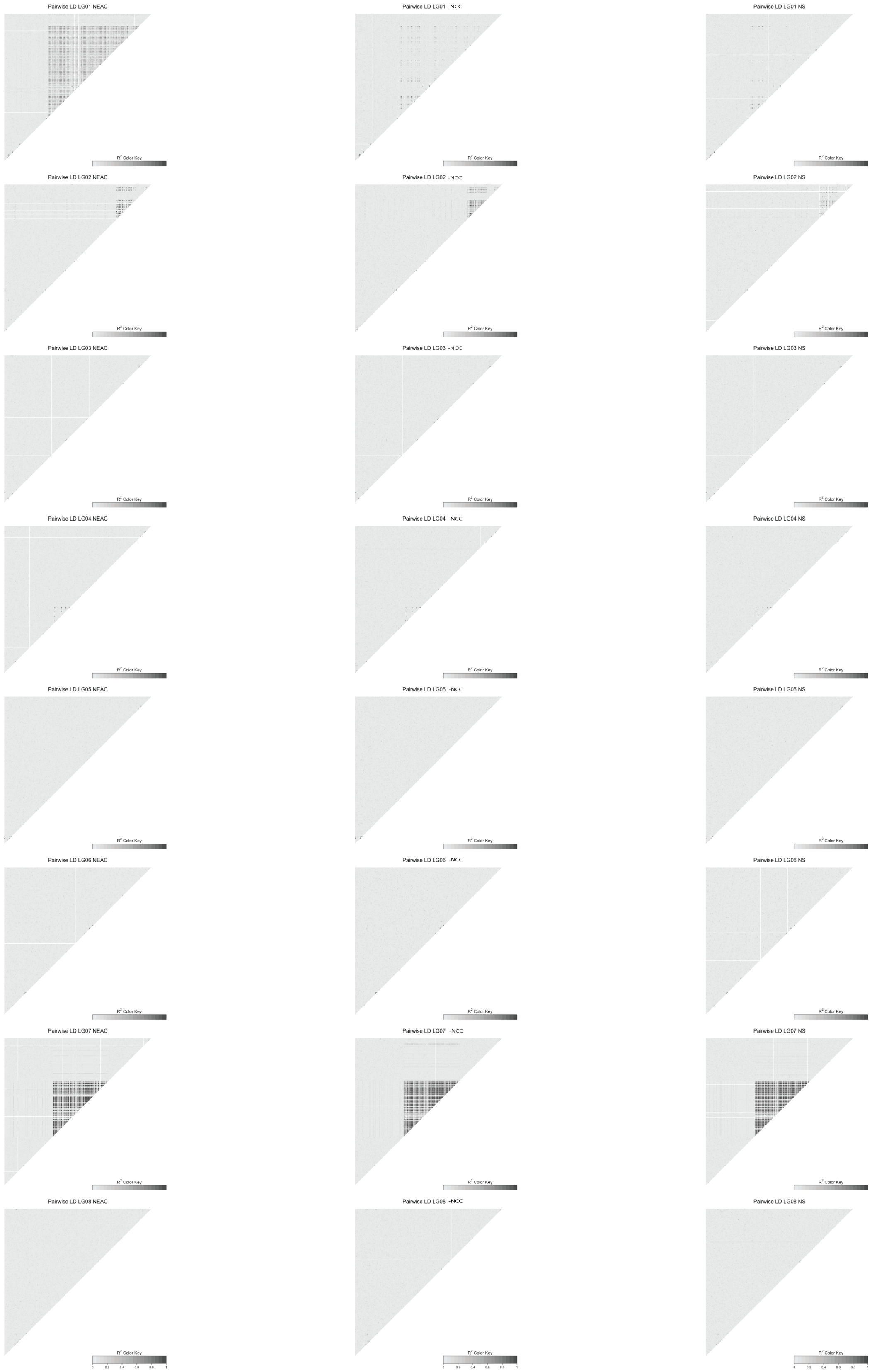
Pairwise LD LG01 NEAC
Pairwise LD LG01 -NCC
Pairwise LD LG01 NS
Pairwise LD LG02 NEAC
Pairwise LD LG02 -NCC
Pairwise LD LG02 NS
Pairwise LD LG03 NEAC
Pairwise LD LG03 -NCC
Pairwise LD LG03 NS
Pairwise LD LG04 NEAC
Pairwise LD LG04 -NCC
Pairwise LD LG04 NS
Pairwise LD LG05 NEAC
Pairwise LD LG05 -NCC
Pairwise LD LG05 NS
Pairwise LD LG06 NEAC
Pairwise LD LG06 -NCC
Pairwise LD LG06 NS
Pairwise LD LG07 NEAC
Pairwise LD LG07 -NCC
Pairwise LD LG07 NS
Pairwise LD LG08 NEAC
Pairwise LD LG08 -NCC
Pairwise LD LG08 NS
R^2 Color Key
0
0.2
0.4
0.6
0.8
1

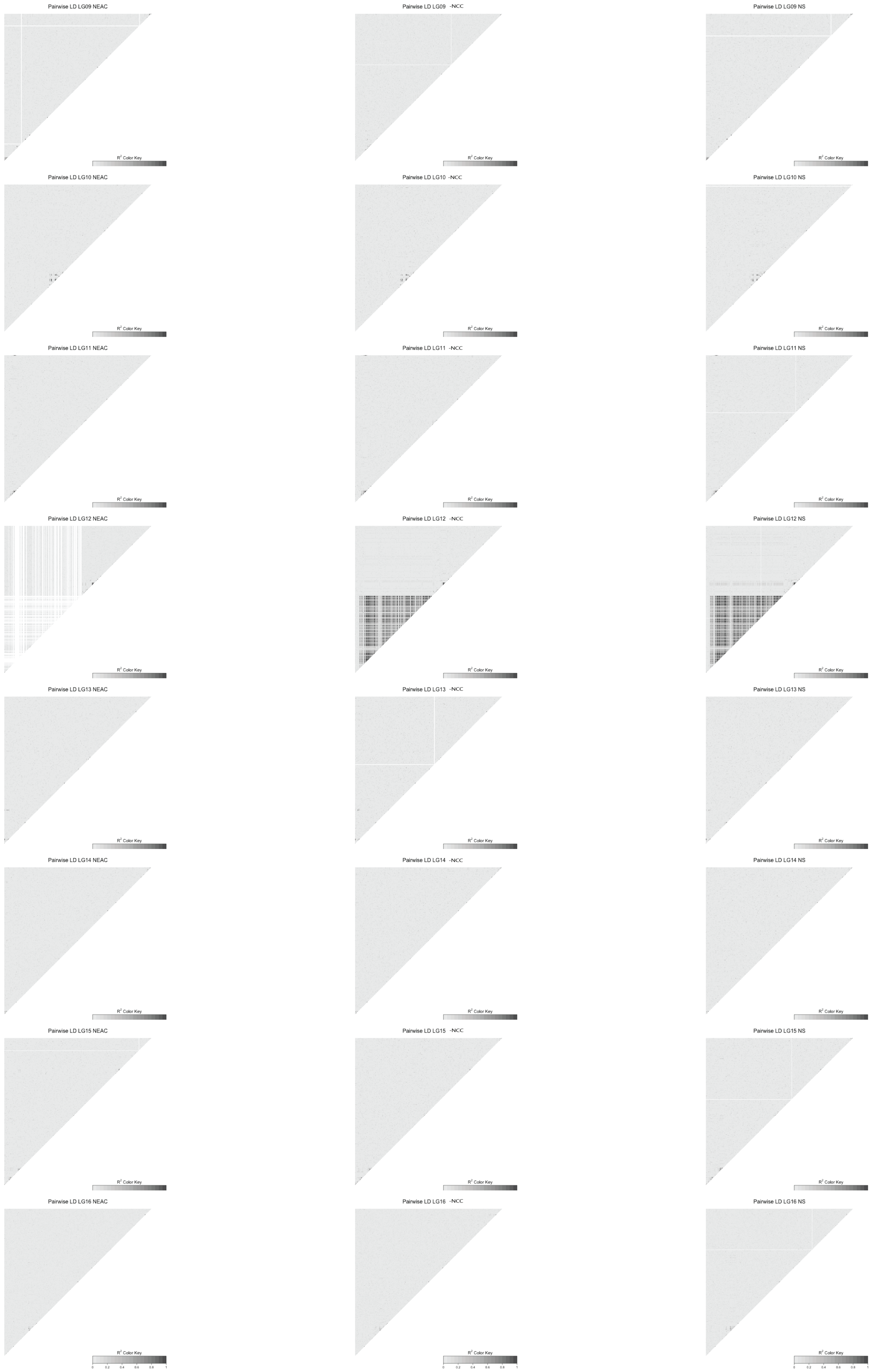

Pairwise LD LG09 NEAC
R2 Color Key
Pairwise LD LG09 -NCC
R2 Color Key
Pairwise LD LG09 NS
R2 Color Key
Pairwise LD LG10 NEAC
R2 Color Key
Pairwise LD LG10 -NCC
R2 Color Key
Pairwise LD LG10 NS
R2 Color Key
Pairwise LD LG11 NEAC
R2 Color Key
Pairwise LD LG11 -NCC
R2 Color Key
Pairwise LD LG11 NS
R2 Color Key
Pairwise LD LG12 NEAC
R2 Color Key
Pairwise LD LG12 -NCC
R2 Color Key
Pairwise LD LG12 NS
R2 Color Key
Pairwise LD LG13 NEAC
R2 Color Key
Pairwise LD LG13 -NCC
R2 Color Key
Pairwise LD LG13 NS
R2 Color Key
Pairwise LD LG14 NEAC
R2 Color Key
Pairwise LD LG14 -NCC
R2 Color Key
Pairwise LD LG14 NS
R2 Color Key
Pairwise LD LG15 NEAC
R2 Color Key
Pairwise LD LG15 -NCC
R2 Color Key
Pairwise LD LG15 NS
R2 Color Key
Pairwise LD LG16 NEAC
R2 Color Key
0 0.2 0.4 0.6 0.8 1
Pairwise LD LG16 -NCC
R2 Color Key
0 0.2 0.4 0.6 0.8 1
Pairwise LD LG16 NS
R2 Color Key
0 0.2 0.4 0.6 0.8 1

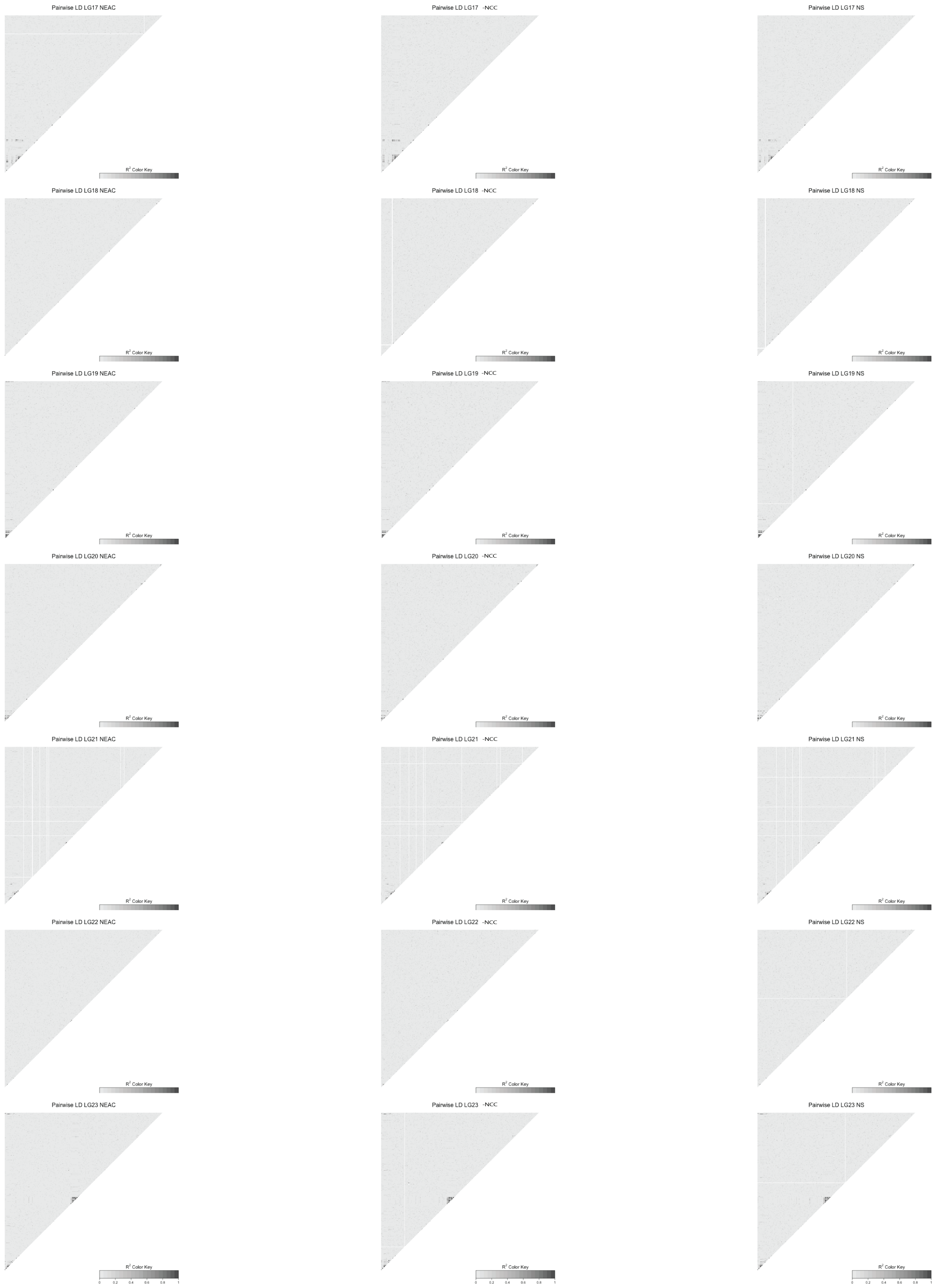

Supplementary Figure S3. **Linkage disequilibrium in all linkage groups.**
Pair-wise LD among loci, measured as r^2, estimated within the NEAC, NCC and NS populations.

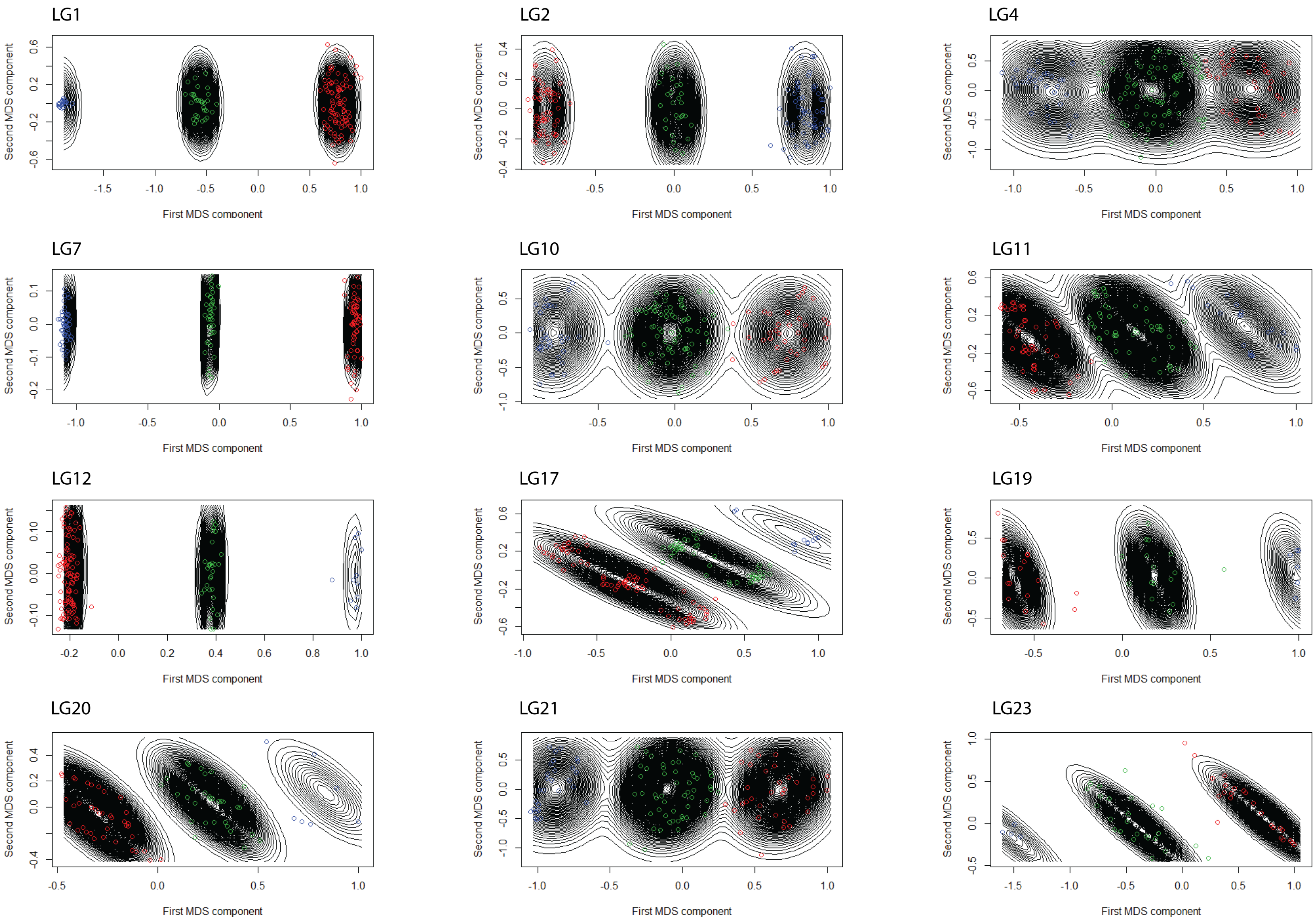

Supplementary Figure S4. **MDS plot showing the inversions detected by InvClust[62].**
The first two MDS components shows a clear three-striped pattern, characteristic of an inversion for these linkage groups. Red and blue circles represent the respective homozygotes while the green circles represent the heterozygotes. Population frequencies are reported in Table 3.

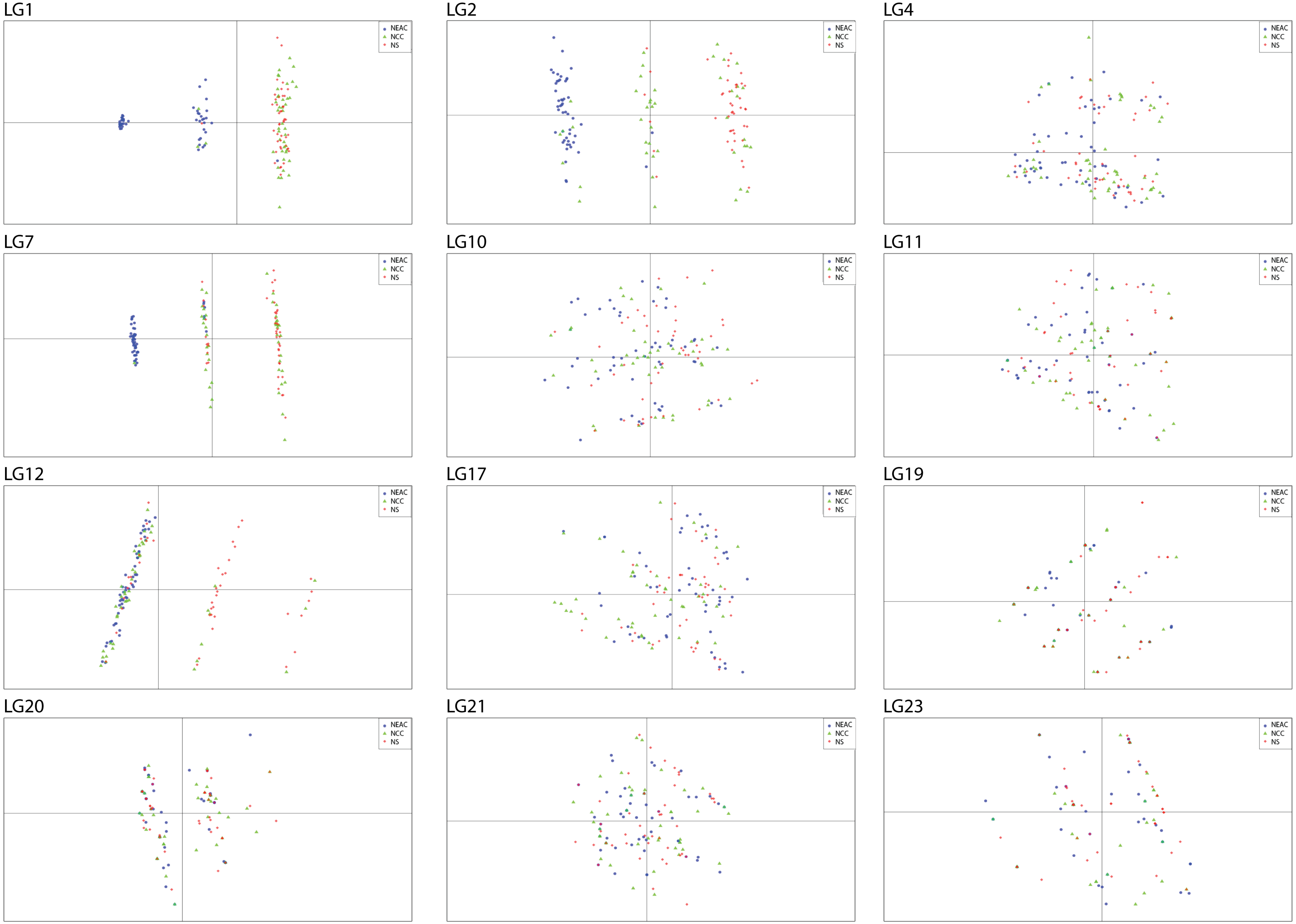

Supplementary Figure S5. **The population structuring within the rearranged regions in Atlantic cod.**
The first two prinsipal components obtained from PCA of the NEAC, NCC and NS populations, using markers within the rearranged regions in the respective LGs.
Each dot represents an individual and the left and right hand clusters represents the homozygotes while the middle cluster represents the heterozygotes.
Population frequencies are reported in Table 3.

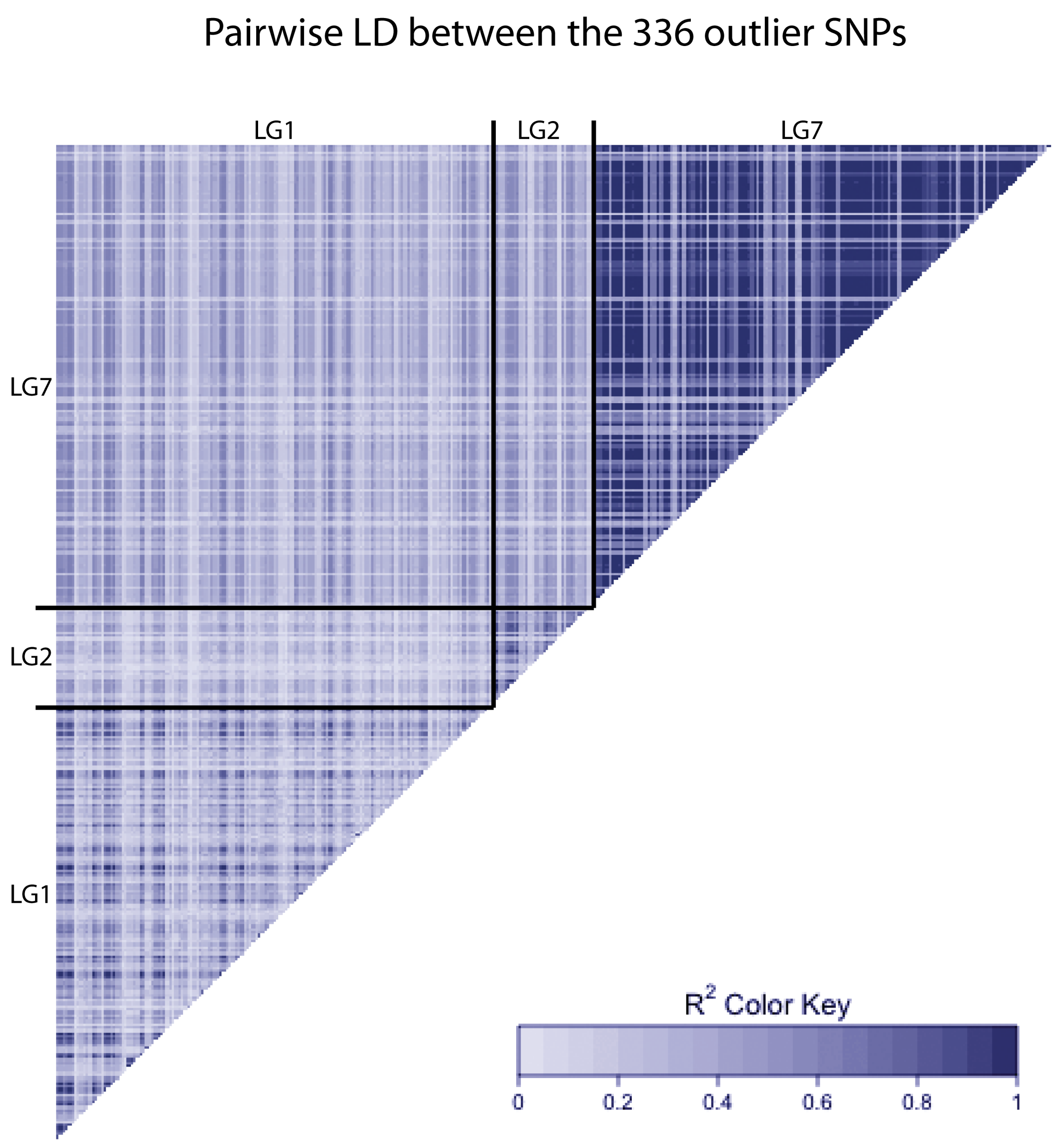

Supplementary Figure S6. **Linkage disequilibrium between 336 outlier loci in LG1, 2 and 7 among Northeast-Arctic cod and Norwegian coastal cod.** The figure shows that there are substantial amount of inter-chromosomal LD between the outliers within LG1, 2 and 7 in the NEAC and NCC populations.
